# A novel localization technique for peripheral ground glass opacity using geometric parameters measured on CT images

**DOI:** 10.1186/s12893-021-01343-8

**Published:** 2021-09-18

**Authors:** Mengjun Bie, Xuemin Zhao, Min Zhang, Guang Fu, Mingjian Ge

**Affiliations:** 1 Department of Cardiothoracic Surgery, The First Affiliated Hospital of Chongqing Medical University, Chongqing, 400016 China; 2 Department of Cardiology, The First Branch Hospital, The First Affiliated Hospital of Chongqing Medical University, Chongqing, 400015 China

**Keywords:** Pulmonary nodule, Ground-glass opacity, Localization technique, Wedge resection

## Abstract

**Background:**

Currently no optimal localization technique has been established for localization of ground glass opacity (GGO). We aimed to introduce a localization technique using geometric localization for peripheral GGO.

**Methods:**

We delineated the location of pulmonary GGO using geometric method which was similar with localization of a point in a spatial coordinate system. The localization technique was based on the anatomical landmarkers (ribs or intercostal spaces, capitulum costae and sternocostal joints). The geometric parameters were measured on preoperative CT images and the targeted GGO could be identified intraoperatively according to the parameters. We retrospectively collected the data of the patients with peripheral GGOs which were localized using this method and were wedge resected between June 2019 and July 2020. The efficacy and feasibility of the localization technique were assessed.

**Results:**

There were 93 patients (male 34, median = 55 years) with 108 peripheral GGOs in the study. All the targeted GGOs were successfully wedge resected in the operative field with negative surgical margin at the first attempt. For each GGO, the localization parameters could be measured in 2–4 min (median = 3 min) on CT images before operation, and surgical resection could be completed in 5–10 min (median = 7 min). A total of 106 (98.15%) GGOs achieved sufficient resection margin. No complications and deaths occurred related to the localization and surgical procedure.

**Conclusions:**

The localization technique can achieve satisfactory localization success rate and good safety profile. It can provide an easy-to-use alternative to localize peripheral GGO.

## Introduction

For surgical treatment of cancer, lobectomy is the standard procedure. However, some studies have showed that sublobar resection (wedge resection and segmentectomy) can achieve comparable oncological outcomes with lobectomy for the patients with GGO-dominant clinical stage IA adenocarcinomas [1]. Although anatomical resection like lobectomy or segmentectomy is still the best approach for the treatment of lung cancer, wedge resection is a commonly used operative procedure for surgical treatment of peripheral GGOs with good oncological outcomes [1, 2]. Segmentectomy can achieve adequate surgical margin for the cT1N0M0 non-small cell carcinoma located in the central area of a segment, nevertheless it remains a great challenge to identify targeted segmental bronchus, intrasegmental and intersegmental veins and arteries [3]. During wedge resection, GGOs are usually very difficult to be identified intraoperatively. Failure of the identification of the target GGO intraoperatively may lead to conversion to thoracotomy or lobectomy. Many kinds of localization techniques have been developed to facilitate the identification of the targeted small pulmonary nodules, including CT-guided percutaneous marking and bronchoscopic marking [4, 5]. The popularity and application of electromagnetic navigation bronchoscopy (ENB) has also been growing [6].

There are strengths and drawbacks in each localization technique. And complications related to the preoperative invasive procedures should not be ignored. Notably, it seems that the current localization techniques make the localization process a little bit complicated and necessitate professional skills and special instruments. Hence, it is very necessary to find an alternative to simplify the localization process and surgeons in some institutions without the availability of special instruments could also easily identify the targeted GGO.

Inspired by the coordinate system to specify a location, we considered a possibility to delineate the location of peripheral GGO using three-dimensional parameters based on the anatomical landmarkers of the thorax. And a localization technique was thus developed. After more than 1 year clinical practice, we considered it was helpful to identify the lesions. In this article, we will introduce this localization technique and assess its efficacy and feasibility to localize peripheral GGO nodules for wedge resection.

## Methods

### Patients

The study was approved by the medical ethics committee of the first affiliated hospital of Chongqing Medical University. Written informed consent was obtained from the patients for the surgical procedure and for the medical data to be used in this study. There was no financial support received for this study. No conflicts of interest are declared.

The study included the patients with GGOs: (1) located at the outer third of the lung parenchyma; (2) suspicious-appearing malignancy and no lymph nodes metastasis was indicated by CT (cN0); (3) biopsy proven malignancies; and (4) receiving wedge resection. The surgery indication of GGO at our center was lesion size over 8 mm [7]. For some GGOs suspicious for malignant lesions with the size of smaller than 8 mm, we also sometimes considered performing surgery for those patients in extremely anxiety influencing normal life. For multiple lesions, the GGOs < 8 mm in size were resected synchronously with the main lesion if malignancies were suspected.

The indications for wedge resection at our center were: (1) the GGO was smaller than 20 mm in size at its greatest dimension and the consolidation/tumor ratio was less than 0.25 [8]; (2) sufficient surgical margin could be achieved estimated on the preoperative CT imaging [9]; or (3) patients could not fit enough to undergo segmentectomy and lobectomy. The surgical strategy for each patient was determined in consulting meeting.

**Fig. 1:**
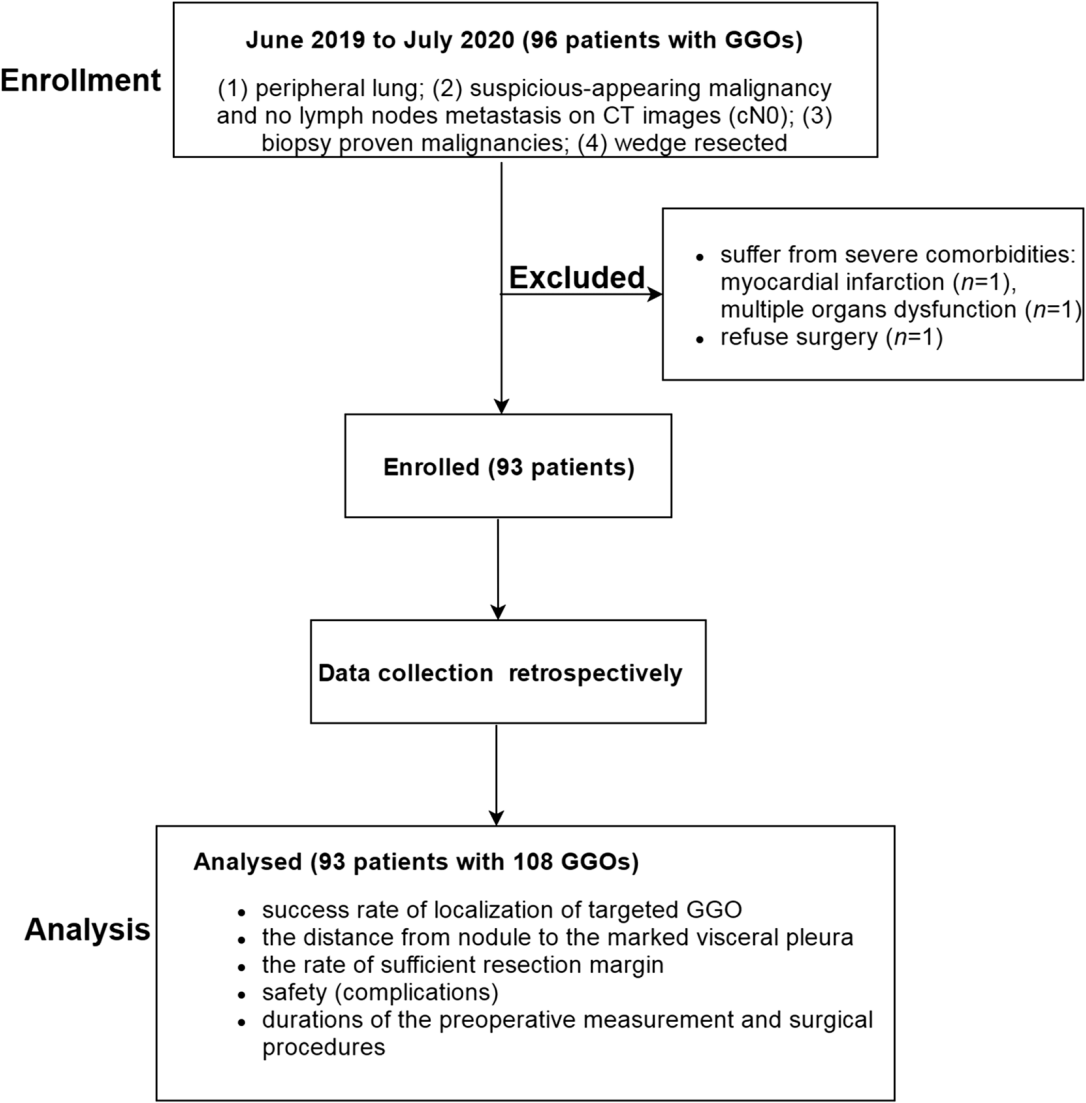
Design of the study

The exclusion criteria included that patients were suffering from severe comorbidities and can’t be tolerable for surgery. From June 2019 to July 2020, a total of 93 patients with 108 peripheral GGOs fulfilled the inclusion criteria and exclusion criteria were enrolled in this study. Figure 1 shows the design of the study.

The power analysis was performed by the PASS V15.0 (Power Analysis and Sample Size, NCSS, LLC. Kaysville, UT). According to the data from literature, the localization success rate was above 98%, and the lower limit of the 95% confidence interval should be higher than 91%^2^. The type I error was 5%. According to the calculation based on these parameters, the power efficacy was 0.88.

### Preoperative measurement

**Fig. 2:**
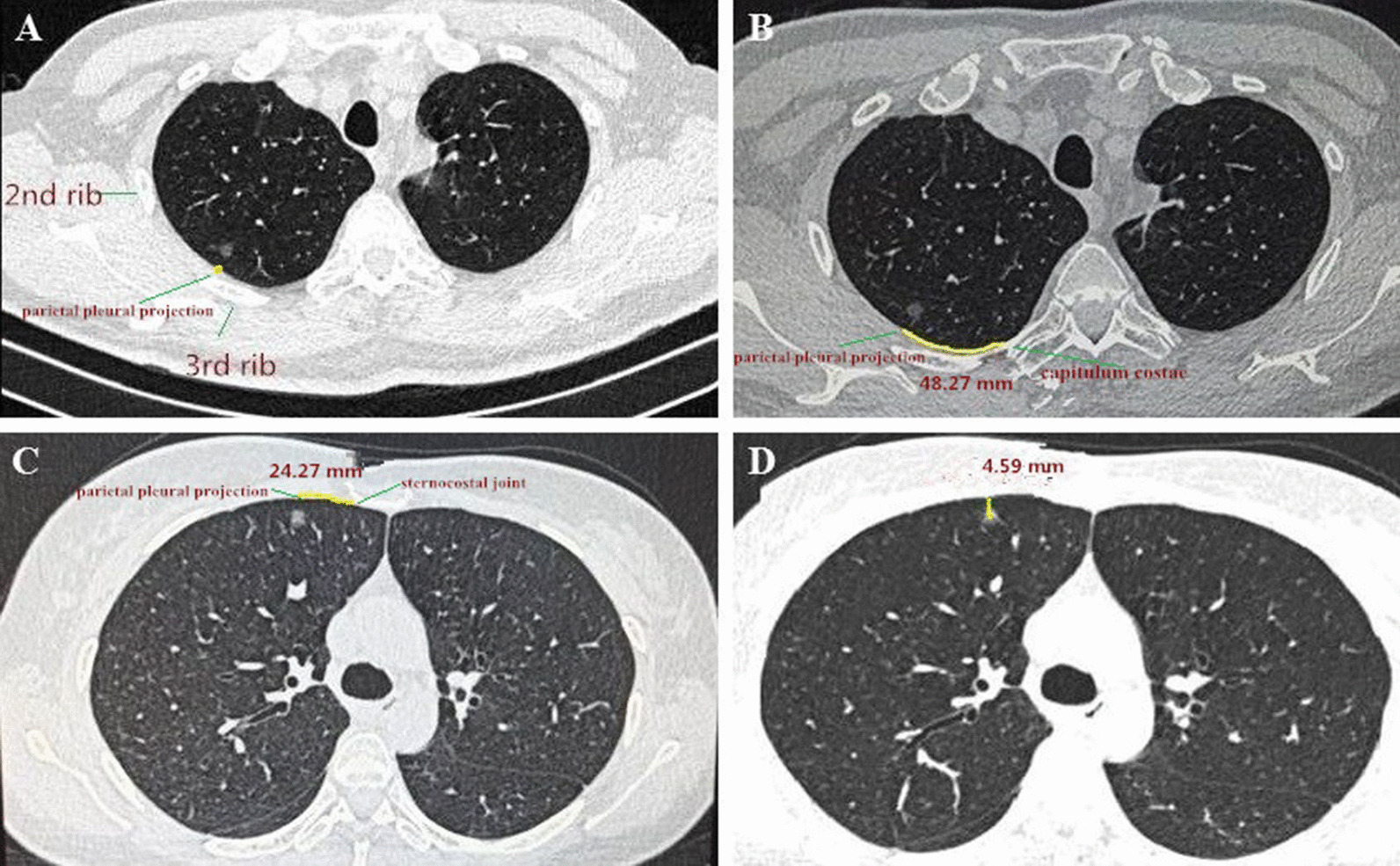
The three-dimensional geometric localization parameters of ground glass opacity (GGO) were measured according to CT imaging series before operation. **A** Y-axis value referred to the rib or intercostal space corresponding to the parietal pleural projection of GGO. **B** For GGO in the rear, X-axis value represented the measured distance between the parietal pleural projection and capitulum costae. **C** For GGO in the front, X-axis value represented the measured distance between the parietal pleural projection and sternocostal joint. **D** Z-axis value was the lesion depth from pleural surface

The localization process included preoperative measurement on CT images and intraoperative identification of the targeted GGO according to the preoperative measurement. The measurement was performed by two experienced surgeon respectively on the day before surgery and consensus was reached after discussion when there were discrepancies. There were three geometric parameters measured on CT images to estimate the localization of GGO (Fig. 2). In the transverse section of CT images, we firstly identify the parietal pleural projection of GGO which was defined as the vertical projection point of the center of the lesion to parietal pleura on CT images. Y-axis value referred to the rib or intercostal space corresponding to the parietal pleural projection (Fig. 2A). X-axis value represented the curvilinear distance between the parietal pleural projection and the landmark-capitulum costae (Fig. 2B) or sternocostal joints (Fig. 2C) measured along the pleural surface. Generally, capitulum costa was preferred as the landmark for the GGO in the rear, and sternocostal joint was preferred for the GGO in the front. Values in X and Y axes could describe the estimated position of parietal pleural projection of GGO. Z-axis value was the lesion depth from pleural surface which was used to estimate the depth of resection (Fig. 2D).

### Surgical procedures

All surgical procedures were performed by three experienced thoracic surgeons in our hospital. Patients were placed in either a left or right lateral decubitus position after dual-lumen endotracheal intubation. The three-port technique for video assistant thoracoscopic surgery (VATS) was preferred. The first port (30-mm) was on the third or fourth intercostal space of the anterior axillary line, as a utility port. The other two ports (20-mm) were all inserted through the seventh intercostal space (midaxillary line and posterior axillary line). Palpation was always the preferred first choice to identify the nodule. Once the nodule was not identified by palpation, this technique will help a lot.

**Fig. 3:**
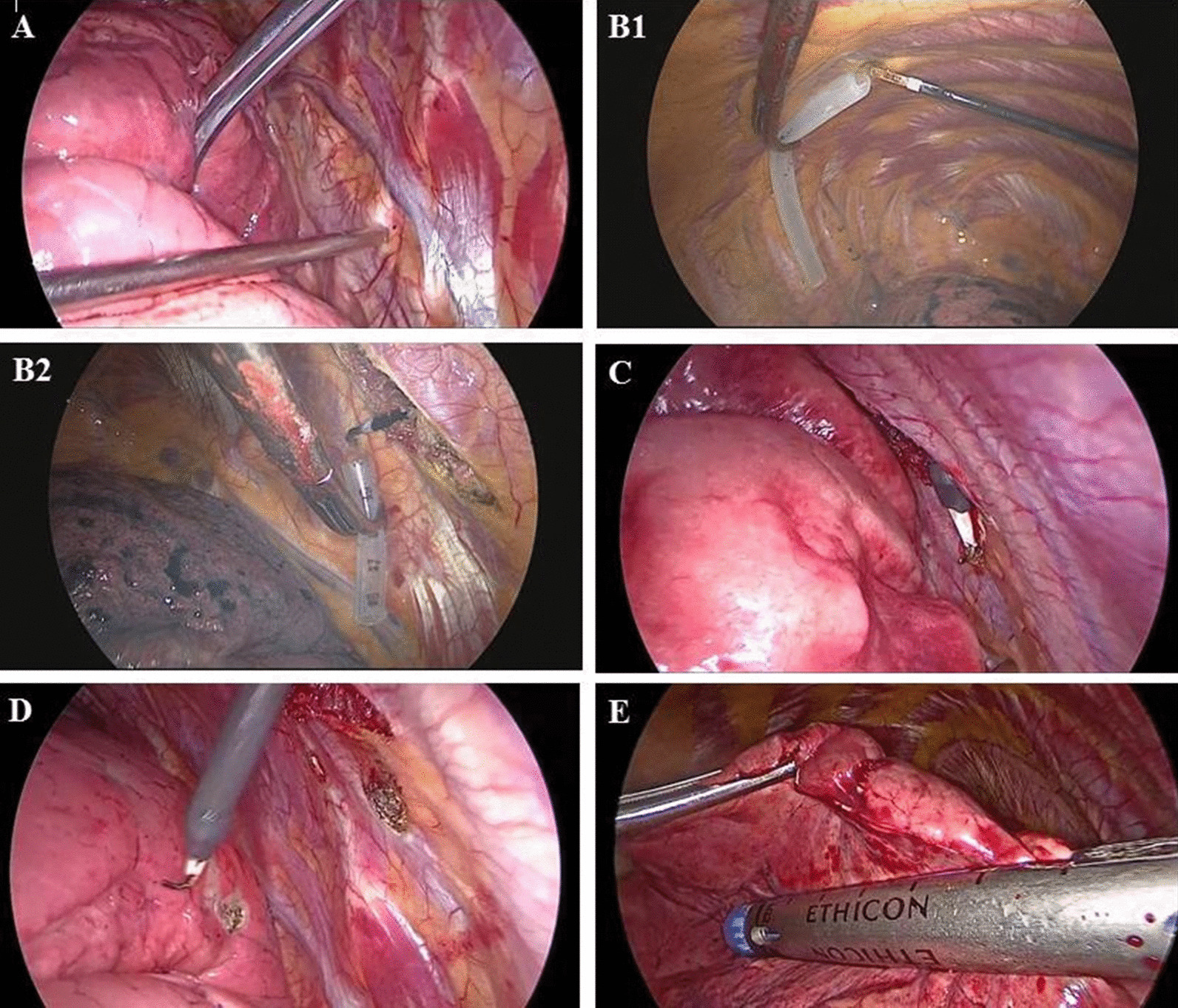
The surgical procedures for wedge resection of GGO. **A** The corresponding rib or intercostal space was firstly identified according to Y-axis value. **B1** We used a thoracic drainage tube at the length of X-axis value for the GGO in the rear. Along the identified rib or intercostal space, we placed the thoracic drainage tube from the sympathetic chain and marked the parietal pleura projection of GGO using electrotome cautery. **B2** For the GGO in the front, the length of the drainage tube was calculated as X-axis value—0.5 cm. We placed the thoracic drainage tube from the internal thoracic vessels and marked the parietal pleura projection using electrotome cautery. **C** Electrotome was placed on the marked parietal pleural projection. And the anesthetist inflated the affected side lung. **D** When the affected side lung was fully inflated, electrotome cautery marked the visceral pleural surface corresponding to the parietal pleura projection. **E** Wedge resection was performed using a linear cutting stapler along the identified rib or intercostal space. The depth of the resection was estimated according to the Z-axis value to guarantee the sufficient resection margin

Firstly, we marked the parietal pleura projection of GGO using the electrotome cautery according to X and Y axes values. The affected lung was deflated before the pleural space was opened. After entering the thorax, the rib was firstly calculated from the top of chest cavity to the below and then the corresponding rib or intercostal space was identified according to Y-axis value (i.e., 4th rib) (Fig. 3A). In chest cavity, we adopted the sympathetic chain and internal thoracic vessels as the landmarks for the GGO in the rear and the GGO in the front respectively. They are easily recognized during surgery. The sympathetic chain is almost at the same level with the capitulum costae and the internal thoracic vessels are about 0.5 cm away from the sternocostal joint. So we use a thoracic drainage tube with scales and cut it at the length as calculated (= X-axis value for the GGO in the rear, and = X-axis value − 0.5 cm for the GGO in the front). We placed the thoracic drainage tube from the landmark along the identified rib or intercostal space and then the parietal pleural projection of GGO could be determined (Fig. 3B1–B2).

Secondly, we marked the visceral pleura corresponding to the marked parietal pleural projection. Electrocautery was placed on the marked parietal pleural projection and the anesthetist was asked to inflate the affected side lung mimicking the deep respiratory state at the time of CT examination (Fig. 3C). When the affected side lung was completely inflated, a burn was made on the visceral pleura opposite the parietal mark (Fig. 3D). Then, the affected lung was deflated again.

Thirdly, the resected pulmonary area was planned according to the marked visceral pleura and the Z-axis value. Wedge resection was performed using a linear cutting stapler (Fig. 3E). The linear cutting stapler was placed underneath the visceral mark, holding pulmonary tissues of approximately at least 2 cm in diameter centering the mark. After the wedge resection was performed, the operative field was cut open around the marked visceral pleura to find the targeted GGO. Intraoperative frozen section examination was performed for the lesions to determine the pathologic patterns and ensure the negative surgical margin. Sufficient resection margin should be guaranteed, or lager wedge resection should be performed. Pathologic characteristics were described by postoperative paraffin section examination and immunohistochemical analysis.

### Data collection

Data were retrospectively collected from the database for demographic characteristics, GGO features like location, size, number; preoperative measurements on CT imaging series; intraoperative data including duration of operation, successful resection of targeted GGO, the distance from nodules to the marked visceral pleura, and sufficient resection margin; complications; and pathological characteristics. Sufficient resection margin was defined as resection margin ≥ 2 cm or the tumor diameter.

### Evaluation

The successful localization procedure was defined as a successful localization of the targeted GGO in the operative field and the success rate of localization of targeted GGO was calculated as follows: [(number of successful localization of targeted GGOs − number of misses of GGOs in the operative field)/number of all GGOs localized] * 100. The distance from nodules to the marked visceral pleura was used to assess the accuracy to localize the targeted GGO. The rate of sufficient resection margin, safety, and durations of the preoperative measurement and surgical procedures were also evaluated. The durations of surgical procedures were measured from the start of intraoperative identification of the targeted GGOs to the removal of the lesions. It didn’t include the time of synchronous lobectomy or segmentectomy, and opening and closing of the incision.

### Statistical analysis

The quantitative data were presented as mean ± standard deviation or a number, median and range. Categorical variable was described as a number and percentage. Univariate logistic and linear regression analyses were performed to determine the associations of GGO related characteristics with sufficient resection margin and the distance from nodules to the marked visceral pleura. Baseline characteristics for univariate analysis include lesion size, lesion type, lesion location, and geometric parameters for GGO. Statistically significant variables (i.e., *P* < 0.20) found by univariate regression analysis were included in the multivariate regression analysis. Two-tailed *P* value < 0.05 was considered to be statistically significant. All statistical analysis was performed using the SPSS 21.0 software for windows (SPSS, Chicago, IL, USA).

## Results

The mean age was 54.0 ± 13.0 years, ranging from 20 to 81 years; 34 (36.5%) patients were male. The baseline demographic and clinical characteristics of the patients are summarized in Table 1. Eleven (11.83%) patients received a contralateral pulmonary operation previously, including 1 patient with wedge resection, 4 patients with segmentectomy and 6 patients with lobectomy.

**Table 1:** The baseline demographic and clinical characteristics of the patients with peripheral ground glass opacity for localization

Variables	Patients (*n* = 93)
Age (years)	54.0 ± 13.0
Median, range	55, 20–81
Sex, n (%)	
Male	34 (37)
Female	59 (63)
Body mass index	23.2 ± 2.88
Smoking history (current/former), n (%)	25 (27)
Family history of lung cancer, n (%)	7 (8)
Comorbidities, n (%)	
Hypertension	15 (16)
Diabetes	6 (7)
Heart disease	4 (4)
Chronic obstructive pulmonary disease	6 (7)
Previous contralateral pulmonary operation, n (%)	11 (12)
History of other malignancies, n (%)	
Colorectal cancer	2 (2)
Breast cancer	2 (2)
Ovarian cancer	1 (1)

Twelve patients had multiple GGOs, and the maximal number of GGO in one patient was three. GGO was classified as pure GGO and mixed GGO. Table 2 showed the clinical characteristics and pathological outcomes of the GGOs. For each GGO, the localization parameters could be measured in 2–4 min (median = 3 min) on CT images before operation. All GGOs underwent wedge resection in VATS, and surgical resection could be completed in 5–10 min (median = 7 min). No procedures were converted to thoracotomy, lobectomy or segmentectomy. All the targeted GGOs were successfully resected in the operative field with negative surgical margin at the first attempt (Table 3), which were then immediately found and confirmed by pathologists.

**Table 2:** Clinical and pathological characteristics of the GGOs for localization

Characteristics	GGOs (*n* = 108)
GGO size (mm)	8.55 ± 2.60
GGO type, n (%)	
Pure GGO	47 (44)
Mixed GGO	61 (57)
GGO location, n (%)	
Right upper lobe	40 (37)
Right middle lobe	8 (7)
Right lower lobe	25 (23)
Left upper lobe	20 (19)
Left lower lobe	15 (14)
Localization parameters	
X-axis value (cm)	8.76 ± 5.57
Y-axis value (median, range)	5th rib, 1st intercostal space-12th rib
Z-axis value (cm)	0.49 ± 0.60
Pathological diagnosis, n (%)	
Atypical adenomatous hyperplasia	8 (7)
Other Benign lesions	4 (4)
Adenocarcinoma in situ	22 (20)
Minimally invasive adenocarcinoma	51 (47)
Invasive adenocarcinoma	23 (21)
Stage, n (%)	
Benign	12(11)
0	22 (20)
IA1	62 (57)
IA2	12 (11)

X-axis value: the measured distance between the parietal pleural projection of GGO and landmark (capitulum costae or sternocostal joint) on CT images; Y-axis value: Y-axis value referred to the rib or intercostal space corresponding to the parietal pleural projection; Z-axis value: lesion depth from pleura; Stage was determined by the eighth edition of the tumor, node, metastasis classification of lung cancer

*GGO* ground glass opacity

**Table 3:** Localization and surgical procedures related characteristics

Variables	Patients (*n* = 93)	GGOs (*n* = 108)
Successful localization rates, n (%)	93 (100)	108 (100)
Durations of preoperative measurement (minutes, median, range)	3, 2–10	3, 2–4
Durations of surgical procedure (minutes, median, range)	9, 5–32	7, 5–10
Pathologically negative surgical margin, n (%)	93 (100)	108 (100)
Sufficient surgical margin, n (%)	91 (98)	106 (98)
The distance from nodule to the marked visceral pleura (cm, median, range)	–	0.6, 0–1.5
Surgical procedures, n (%)		
Bilateral pulmonary operation	8 (9)	–
Unilateral pulmonary operation for multiple lesions	13 (14)	–
Multiple wedge resections	10 (11)	–
Wedge resection and segmentectomy	8 (9)	–
Wedge resection and lobectomy	7 (8)	–
Pleural adhesion, n (%)	6 (7)	–
No placement of thoracic drainage tube	8 (9)	–

Sufficient resection margin was defined as resection margin ≥ 2 cm or the tumor diameter

The median distance from nodules to the marked visceral pleura was 0.6 cm (0–1.5 cm). A total of 106 (98.15%) GGOs achieved sufficient resection margin. Only 2 pure GGOs had a shortage of resection margin, although they were completely removed with a pathologically negative surgical margin. The lesion depths for the two GGOs were 2.4 cm and 2.6 cm respectively. Enlarged wedge resection was performed to guarantee the sufficient resection margin. No complications and deaths occurred related to the localization and surgical procedure.

In univariate analysis (Table 4), the median distance from nodules to the marked visceral pleura was only significantly associated with X-axis value (Standard coefficient = 0.061, *P* = 0.000), and sufficient surgical margin was only significantly associated with Z-axis value (OR = 12.204, *P* = 0.012). Multivariate regression analysis was not performed because other characteristics related to GGOs were not significantly indicated in univariate analysis.

**Table 4:** A univariate linear regression analysis of the GGO related characteristics associated with the distance from nodule to the marked visceral pleura and a univariate logistic regression analysis of the GGO related characteristics associated with the sufficient resection margin

	Univariate linear regression analysis (The distance from nodule to the marked visceral pleura)			Univariate logistic regression analysis (sufficient resection margin)		
	Coefficient	*t*	*P*	Odds ratio	95% CI	*P*
Lesion size	0.008	0.971	0.334	0.969	0.678,1.385	0.864
Lesion type	− 0.037	− 0.503	0.616	–	–	0.998
Lesion location	0.007	0.303	0.762	0.932	0.356,2.438	0.886
X-axis value	0.061	21.233	0.000	0.875	0.638,1.199	0.405
Y-axis value	− 0.002	− 0.108	0.914	1.216	0.714,2.071	0.471
Z-axis value	− 0.027	− 0.450	0.653	12.204	1.749,85.137	0.012

X-axis value: the measured distance between the parietal pleural projection of GGO and landmark (capitulum costae or sternocostal joint) on CT images; Y-axis value: Y-axis value referred to the rib or intercostal space corresponding to the parietal pleural projection; Z-axis value: lesion depth from pleura

*P* < 0.20 was considered as significant which would be included in the multivariate regression analysis

*CI* confidence interval

## Discussion

This study has shown a high successful localization rate and good safety profile of the localization technique for peripheral GGO in wedge resection. It can be considered as an effective and safe method in localizing the peripheral GGOs. It can provide an easy-to-use alternative to localize peripheral GGO without assistance with the professional skills and special instruments.

Some localization techniques and effective devices have been developed to help identify the targeted GGO with a high successful localization rate, such as hookwire localization, microcoil localization, methylene-blue and ENB [10–12]. However, they need the assistance of special devices and professional skills, and they are not available in a lot of institutions. Current localization techniques seem complicate the process of preoperative preparation, resulting in increased medical costs and patients’ waiting time. The new localization method can be an important alternative especially when other localization techniques can’t be available. It can also simplify the localization process for the pulmonary nodules to improve work efficiency. The surgeons can undergo the localization process by themselves without multi-disciplinary collaboration and assistance of professional skills and special devices.

The underlying principle of this technique was to localize the lesion using three-dimensional parameters based on the anatomical landmarkers of the thorax. These landmarkers could not be shifted as position changed. We used only one landmarker in latitudinal line for preoperative measurement-capitulum costae or sternocostal joints, which were easily recognized. And it was very easy to localize the parietal pleural projection according to the X-axis value. As we know, it was much different from the previous reported techniques using the anatomical landmarkers [13, 14]. Our results showed that all GGOs were successfully resected in the operative field. The successful localization rate of the localization technique was similar to the commonly used localization techniques reported. Notably, our localization technique could be accessible to the areas which a CT-guided percutaneous approach cannot reach, including lung apex, and those facing mediastinum, diaphragm and scapula. Moreover, this method could be considered to be safe. Our results showed that this technique was free of any complications related to the localization process. With the application of this method, preoperative localization of the pulmonary nodule could be completed just using the CT images. Patients didn’t have to receive an additional invasive procedure before pulmonary operation, and didn’t have the chest discomfort due to the percutaneous puncture or bronchoscopy; hence better feelings could be experienced.

Wedge resection is not an anatomically procedure like the lobectomy and segmentectomy, the resection range is usually not clearly defined and tends to be subjective with poor reproducibility. The difficulty in estimating the resection depth also leads to the concern about the sufficient resection margin. The sufficient resection margin should be guaranteed to prevent local recurrence after operation [15]. This localization technique could partly solve these problems based on the measured parameters. We planned the resection line along the direction of the identified rib or intercostal space (Y-axis value) and estimated the resection depth according to the lesion depth from pleura (Z-axis value). Our experience suggested that sufficient resection margin tended to be easily acquired for the nodules located in outer third of lung field, especially for those close to pulmonary surface. As the lesion seated deeper, the acquisition of the sufficient resection margin seemed to be increasingly difficult. It was similar to CT-guided percutaneous marking and virtual-assistant lung mapping. Masahiro Yanagiya et al. reported that the optimal cutoff value of the required resection depth was 3.1 cm [16]. We evaluated the accuracy of localization using the distance from nodules to the marked visceral pleura. Our results were satisfactory. The median distance was 0.6 cm (ranging from 0 to 1.5 cm), and the distance from nodules to the marked visceral pleura was within 1 cm for 88.89% of the GGOs. It was only associated with the X-axis value indicated by the linear regression analysis. Our experience suggested that satisfactory accuracy of localization could be achieved when the distance was within 10 cm between the parietal pleural projection and the specified landmark-capitulum costae or sternocostal joint (X-axis value ≤ 10 cm). For the GGO of which X-axis value was more than 10 cm, the bias was a little bigger but still could be acceptable.

There were several aspects to explain the bias. The obliquity of the direction of ribs led to the measuring bias. The farther the parietal pleural projection was away from specified landmark, the bigger the measurement bias was. As the X-axis value > 10 cm, the difficulty turned to be increased to operate the thoracic drainage tube in the chest cavity and recognized the right rib covered by the intercostal muscle. Additionally, the respiratory state, the respiratory movement and change of the position might also have an influence on the localization accuracy of the targeted GGO.

This technique is easy to operate and does not need a lot of time. For each GGO, the localization parameters could be measured in 2–4 min (median = 3 min) on CT images before operation, and surgical resection could be completed in 5–10 min (median = 7 min). Additionally, there were no additional costs for the patients. We suggest that the patients should be selected carefully so that wedge resection can achieve good oncological outcomes. The GGO should be smaller than 20 mm in size at its greatest dimension and the consolidation/tumor ratio was less than 0.25 [8]. And the lesion should be located in the outer third of lung field in order to guarantee sufficient surgical margin. We suggest that the resection area should be more than 2 cm of lung tissues centering mark on the visceral pleura. For the GGO of which X-axis value was more than 10 cm and that located nearby the free margin of inferior lobe, lager pulmonary areas should be resected due to the lower accuracy of localization.

There were several limitations in this study. Our study was a single-center design, and the sample size was relatively small. We didn’t compare the new localization technique with the currently used localization techniques. We will further improve this technique to make it more accurate, especially for the nodules with X-axis value > 10 cm. We hope to establish a model to promote its application in different institutions.

## Conclusions

This localization technique can achieve satisfactory localization success rate and avoid some complications. It can provide an easy-to-use alternative to localize peripheral GGO. Additionally, it can simplify the process of localization for GGO.

## Data Availability

The datasets used and/or analyzed in this present study are available from the corresponding author on reasonable request.

## Abbreviations

GGO: Ground glass opacity
ENB: Electromagnetic navigation bronchoscopy
VATS: Video assistant thoracoscopic surgery
